# A severe microsporidian disease in cultured Atlantic Bluefin Tuna (*Thunnus thynnus*)

**DOI:** 10.1186/s43008-022-00090-6

**Published:** 2022-03-11

**Authors:** Alejandro López-Verdejo, Francisco E. Montero, Fernando de la Gándara, Miguel A. Gallego, Aurelio Ortega, Juan Antonio Raga, José F. Palacios-Abella

**Affiliations:** 1 Marine Zoology Unit, Cavanilles Institute of Biodiversity and Evolutionary Biology, Science Park, University of Valencia, C/ Catedrático José Beltrán 2, 46980 Paterna, Spain; 2 Instituto Español de Oceanografía, Centro Oceanográfico de Murcia, 30860 Puerto de Mazarrón, Murcia, Spain

**Keywords:** *Microsporidia*, *Marinosporidia*, *Glugeida*, *Glugea thunni* new taxa, Aquaculture, *Osteichthyes*, *Scombridae*, Xenoma

## Abstract

One of the most promising aquaculture species is the Atlantic bluefin tuna (*Thunnus thynnus*) with high market value; disease control is crucial to prevent and reduce mortality and monetary losses. *Microsporidia* (*Fungi*) are a potential source of damage to bluefin tuna aquaculture. A new microsporidian species is described from farmed bluefin tunas from the Spanish Mediterranean. This new pathogen is described in a juvenile associated with a highly severe pathology of the visceral cavity. Whitish xenomas from this microsporidian species were mostly located at the caecal mass and ranged from 0.2 to 7.5 mm. Light and transmission electron microscopy of the spores revealed mature spores with an average size of 2.2 × 3.9 μm in size and a polar filament with 13–14 coils arranged in one single layer. Phylogenetic analysis clustered this species with the *Glugea* spp. clade. The morphological characteristics and molecular comparison confirm that this is a novel microsporidian species, *Glugea thunni*. The direct life-cycle and the severe pathologies observed makes this parasite a hard risk for bluefin tuna cultures.

## INTRODUCTION

Common fish diseases known as “fungal” are mostly produced by not real fungi organisms as oomycetes. These diseases are usually external and only a few can affect internal organs (Woo and Bruno 2011). Only a few species of ascomycetes, as *Branchyomyces* spp. have been recently associated to diseases in fish cultures (El-Sayed, 2020). *Microsporidia* are known for more than 100 years; first described species was *Nosema bombicys*, pathogen of silkworms (Pasteur, 1870). These parasites were not originally considered as fungi since molecular evidence showed their close phylogenetical relationship and finally microsporidian were included among them (Thomas et al. 1996; Hirt et al. 1999; Van de Peer et al. 2000). In 2006, microsporidians were nomenclaturally recognized as fungi in *The International Code of Botanical Nomenclature (Vienna Code)* in 2005 (McNeill et al. 2006). However, in order to avoid potentially many name changes, in 2011 they were excluded from being subject to the provisions of the *International Code of Botanical Nomenclature*, now the *International Code of Nomenclature for algae*, *fungi*, *and plants* (ICNafp), despite phylogenetically belonging to the kingdom *Fungi* (Redhead et al. 2009; Turland et al. 2018). Names of *Microsporidia* consequently continue to be governed by the rules of the *International Code of Zoological Nomenclature* (ICZN) (ICZN 1999).

*Microsporidia* can be found in almost any environment and can infect large numbers of vertebrate and invertebrate species (Kent et al. 2014). They are obligate intracellular parasites that are able to infect a wide variety of hosts, including many species of fish (Mathis et al. 2005). Currently, 18 genera of microsporidians infecting fish have been described (Azevedo et al. 2016). Some are considered a real issue in aquaculture for causing major diseases and mortality of the fish which consequently has a negative economic impact (Bulla and Cheng 1976; Kent et al. 2014; Ryan and Kohler 2016).

One of the main issues for the management of fish cultures is disease control, often subjected to new pathologies related to unknown pathogens (Woo 2006). The Atlantic bluefin tuna (ABT, *Thunnus thynnus*) is one of the most promising new species in Mediterranean aquaculture, due to its large size, fast growth and high market value. In fact, bluefin tunas (*Thunnus* spp.) are some of the highest market value fishes worldwide (FAO 2018, 2020). The culture of this species is still based on the fattening of juveniles captured in the natural environment since, although the closure of the life cycle has been achieved in captivity, no profitable production levels have been achieved yet (De la Gándara et al. 2016; Ortega and De la Gándara 2017; FAO 2018; APROMAR 2020).

A total of 89 different parasites have been reported in ABT (Munday et al. 2003, Mladineo et al. 2011, Mladineo and Lovy 2011, Culurgioni et al. 2014, Palacios-Abella et al. 2015, Rodríguez-Llanos et al. 2015. Among the parasites described in bluefin tunas, the only microsporidian species reported to date are *Microsporidium* sp. and *M. milevae* (Mladineo and Lovy, 2011), infecting the muscle of *T. orientalis* and the intestine of *T. thynnus*, respectively, with no relevant pathologies reported (Zhang et al. 2004; Mladineo and Lovy 2011). In this study a *Glugea*-like microsporidian infecting ABT. *Glugea* is one of the microsporidian genera with one of the highest number of species, with at least 30 species infecting various organs in the fish hosts (Azevedo et al. 2016; Lom 2002; WoRMS 2021).

This study is focused on a new microsporidian disease found in an ABT culture in the Spanish Mediterranean. The microsporidian was associated with severe visceral infection compromising fish survival and possibly causing consumer rejection. The microsporidian and related pathologies are described using morphological and molecular analyses, with the aim of providing diagnostic tools. The possible transmission path is discussed, providing recommendations to avoid this harsh disease.

## MATERIALS AND METHODS

### Host and parasites sampling

Parasites were observed during routine farm check-ups in one dead juvenile ABT. This specimen belonged to an experimental ABT stock born in the Marine Aquaculture Plant of the facilities of Spanish Institute of Oceanography (IEO) located in Mazarrón, (Murcia, SE Spain) from eggs collected from brood fish maintained in sea cages. Larvae were reared in land-based facilities until 45 days (around 10 g wet weight), and then moved to sea cages placed off San Pedro del Pinatar (Murcia, SE Spain). Tunas were fed on thawed bait, mainly European pilchard (*Sardina pilchardus*) and round sardinella (*Sardinella aurita*). Parasites were found in November 2017, when tunas were 5 months old (about 30 cm of total length and 800 g of wet weight). The infected tuna was dissected fresh and inspected with the naked eye. Samples of the infected tissues and encysted xenomas (from now on referred as xenomas) were collected and fixed in both formaldehyde 10% and glutaraldehyde 2.5% in cacodylate buffer 0.1 M (pH 7.4 v/v) for posterior light and electron microscopy analyses, respectively. Some xenomas were also fixed in absolute ethanol for molecular study. Formaldehyde-fixed samples were also examined with a stereomicroscope (Leica MZ6 at 20–40×).

### Microscopy techniques

The isolated xenomas were measured previous to their extraction. The spores were first observed and measured in fresh smear preparations with light microscopy (Leica DMR) at 100 × . Part of the specimens fixed in formaldehyde were embedded in paraffin and cut in 5 μm sections with a Leica RM 2125RT microtome and then stained in hematoxylin–eosin for further observations.

Glutaraldehyde fixed xenomas were cut in semi-thin and ultrathin sections in the Central Service for Experimental Research (SCSIE) of the University of Valencia. Fixed xenomas were washed three times and then postfixed in osmium tetroxide 1% in cacodylate buffer 0.1 M. After washing with the sodium cacodylate, xenomas were dehydrated in increasing concentrations of ethanol and embedded in epoxy resin. Sections were performed in a Leica VT1200S ultramicrotome to obtain semi-thin (2 μm) and ultrathin (60–70 nm) sections. Semi-thin sections were stained in toluidine blue and ultrathin sections (60–70 nm) in uranyl acetate (20 min) and lead citrate (5 min). Images were acquired in the Electron Microscopy Unit of the SCSIE with the Transmission Electron Microscope (JEOL JEM 1010 with AMT RX80 (8Mpx) digital camera) operated at 80 kV. Measurements were obtained from 20 individuals, except when otherwise indicated.

### Molecular data

For the molecular study DNA extraction was performed with the ®Blood & Tissue kit (Quiagen, Venlo, The Netherlands), directly from the excised xenoma (submitted previously to a mechanical rupture process, xenoma were ruptured inside a 1.5 ml sterile Eppendorf using a plastic pestle) and following the manufacturer’s instructions.

The 16S gene of the ribosome was amplified by PCR with the following primer pairs: (V1f (5′-CACCAGGTTGATTCTGCC-3′) with HG5F_rev (5′-TCACCCCACTTGTCGTTA-3′), and HG4F (5′-CGGCTTAATTTGACTCAAC-3′) with HG4R (5′-TCTCCTTGGTCCGTGTTTCAA-3′). The PCR were performed in 20 µl reactions with 3 µl of DNA sample, 1.6 µl of each primer at 5 mM and 10 µl of MyFi Mix (Bioline Ltd., London, United Kingdom). The thermocycling amplification program consisted of a preliminary denaturation step at 94 °C (5 min) followed by 40 cycles of 94 °C (50 s), 50 °C (50 s), 72 °C (2 min) ending with a final extension step at 72 °C (10 min) and then preserved at 4 °C. The amplicons were visualized in a 1% agarose gel with GelRed stain on a ~ 35 min, 95 V electrophoresis.

The sequencing was performed using the same PCR primers and carried out at Macrogen Europe Inc. (Amsterdam, The Netherlands) on a 3730xl DNA Analyzer (Applied Biosystems, Foster City, CA). The obtained sequences were assembled using BioEdit and submitted to the Basic Local Alignment Search Tool (BLAST) on GenBankTM to check for sequence identity.

### Phylogenetic analysis

The newly generated sequence was aligned with available sequences retrieved from GenBank (Table 1). We performed two different alignments varying the sequences used and their length, bearing in mind the limitations imposed by the differences in the length among the sequences of the selected species and the differences of base pairs in the alignment with the shortest sequences (Tables 2, 3). Sequences were aligned with MUSCLE (Edgar 2004) implemented in MEGA v7 (Kumar et al. 2016). Non-homologous regions were removed prior to analyses using Gblocks implemented in SEAVIEW v4.6.1 (Gouy et al. 2010). Neighbour-joining (NJ), maximum likelihood (ML) and Bayesian inference (BI) analyses were used to explore the relationships of *Glugea thunni* in relation to the other available sequenced species of *Glugea*. Neighbour-joining analyses of Kimura-2-parameter distances using 1000 bootstrap resampling used to estimate the nodal support. BI analyses were carried out with MrBayes v 3.2.3 (Ronquist and Huelsenbeck 2003) and ML analyses were performed with PhyML 3.0 (Guindon et al. 2010) with a non-parametric bootstrap validation based on 1000 replicates. The general time-reversible model with gamma distributed among-site rate variations (GTR + Γ) was estimated as the best-fit nucleotide substitution model using jModelTest 2.1 (Guindon and Gascuel 2003; Darriba et al. 2012). Posterior probability distributions were generated using Markov Chain Monte Carlo (MCMC) method. MCMC searches were run for 10.000.000 generations on two simultaneous runs of four chains and sampled every 1.000 generations. The 'burn-in' was set for the first 2.500 sampled trees which were discarded prior to analyses. The trees were visualized with FigTree v 1.4.2 (Rambaut 2012).

**Table 1:** Summary of the sequences of microsporidians used in the phylogenetic analyses retrieved from GenBank

Parasite species	Host species	GenBank accession no	Reference
*Glugea anomala* (Moniez, 1887)	*Gasterosteus aculeatus* L	AF044391	Nilsen et al. (1998)
*Glugea arabica* Azevedo, Abdel-Baki, Rocha, Al-Quraishy and Casal, 2016	*Epinephelus polyphekadion* (Bleeker, 1849)	KT005391	Azevedo et al. (2016)
*Glugea atherinae* Berrebi, 1979	*Atherina boyeri* Risso, 1810	U15987	Da Silva et al. (unpublished data)
*Glugea eda* Mansour, Zhang, Abdel-Haleem, Darwish, Al-Quraishy, Abdel-Baki, 2020	*Caesio striata* Rüppell, 1830	MK568064	Mansour et al. (2020)
*Glugea epinephelusis* Wu, Wu, Wu, 2005	*Epinephelus akaara* (Temminck and Schlegel, 1842)	AY090038	Wu et al. (2005)
*Glugea gasterostei* Voronin, 1974	*Gasterosteus aculeatus* L	KM977990	Tokarev et al. (2015)
*Glugea hertwigi* Weissenberg, 1911	*Osmerus eperlanus eperlanus* (L.)	GQ203287	Lovy et al. (2009)
*Glugea jazanensis* Abdel-Baki, Tamihi, Al-Qahtani, Al-Quraishy, Mansour, 2015	*Lutjanus bohar* (Forsskål, 1775)	KP262018	Abdel-Baki et al. (2015b)
*Glugea nagelia* Abdel-Baki, Al-Quraishy, Rocha, Dkhil, Casal, Azevedo, 2015	*Cephalopholis hemistiktos* (Rüppell, 1830)	KJ802012	Abdel-Baki et al. (2015a)
*Glugea plecoglossi* Strickland, 1911	*Plecoglossus altivelis* (Temminck and Schlegel, 1846)	AJ295326	Bell et al. (2001)
*Glugea sardinellensis* Mansour, Thabet, Harrath, Al Omar, Mukhtar, Sayed, Abdel-Baki, 2016	*Sardinella aurita* (Valenciennes)	KU577431	Mansour et al. (2016)
*Glugea serranus* Casal, Rocha, Costa, Al-Quraishy, Azevedo, 2016	*Serranus atricauda* Günther, 1874	KU363832	Casal et al. (2016)
*Glugea stephani* (Hagenmüller 1899)	*Platichthys flesus* (L.)	AF056015	Pomport-Castillon et al. (unpublished data)
*Glugea thunni *sp. nov	*Thunnus thynnus* (L.)	OM914139	This study
*Loma embiotocia* Shaw, Kent, Docker, Brown, Devlin, Adamson, 1997	*Cymatogaster aggregata* Gibbons, 1854	AF320310	Brown (unpublished data)
*Loma morhua Morrison*, Sprague, 1981	*Gadus morhua* L	GQ121037	Frenette et al. (unpublished data)
*Loma salmonae* (Putz, Hoffman, Dunbar, 1965)	*Oncorhynchus mykiss* (Walbaum, 1792)	U78736	Docker et al. (1997)
*Microgemma caulleryi* Leiro J, Sanmartin M, Iglesias R and Ubeira F, 1999*	*Hyperoplus lanceolatus* (Le Sauvage)	AY033054	Leiro et al. (unpublished data)
*Microgemma* sp. partial 16S	*–*	AJ252952	Cheney et al. (2000)
*Pleistophora ehrenbaumi* Reichenow, 1929	*Anarhichas lupus* L	AF044392	Nilsen et al. (1998)
*Pleistophora mirandellae* Vaney and Conte, 1901	*Rutilus rutilus* (L.)	AJ295327	Bell et al. (2001)
*Pleistophora typicalis Gurley*, *1893*	*Myoxocephalus scorpius* (L.)	AF044387	Nilsen et al. (1998)
*Outgroup*			
*Brachiola algerae* Vavra and Undeen, 1970	*Anopheles stephensi* Liston 1901	AY230191	Coyle et al. (2004)

*Accepted as *Glugea microspora* inLom (2002)

**Table 2:** Differences among representatives of the genera *Brachiola*, *Glugea*, *Loma*, *Microgemma* and *Pleistophora* for 16S rDNA sequences, pairwise nucleotide differences (above the diagonal) and p-distances (below the diagonal) 774 bp sequences

	Species	1	2	3	4	5	6	7	8	9	10	11	12	13	14	15	16	17	18	19	20	21	22	23
1	*Brachiola algerae*	–	206	197	205	200	200	204	205	197	198	204	210	197	205	205	211	213	212	191	188	206	216	210
2	*Glugea anomala*	0.315	–	60	2	58	62	2	2	61	62	1	18	61	4	2	103	89	91	108	118	75	71	78
3	*Glugea arabica*	0.302	0.090	–	58	4	3	58	58	1	1	59	69	1	60	58	106	97	97	117	113	72	70	73
4	*Glugea atherinae*	0.313	0.003	0.086	–	56	60	0	0	59	60	1	16	59	2	0	101	87	89	107	116	74	70	77
5	*Glugea eda*	0.306	0.087	0.006	0.083	–	5	56	56	5	5	57	67	5	58	56	107	97	97	118	116	70	72	71
6	*Glugea epinephelusis*	0.306	0.093	0.004	0.089	0.007	–	60	60	4	4	61	71	4	62	60	110	100	100	120	117	74	74	75
7	*Glugea gasterostei*	0.311	0.003	0.087	0.000	0.083	0.089	–	0	59	60	1	16	59	2	0	101	87	89	106	115	74	70	77
8	*Glugea hertwigi*	0.313	0.003	0.086	0.000	0.083	0.089	0.000	–	59	60	1	16	59	2	0	101	87	89	107	116	74	70	77
9	*Glugea jazanensis*	0.301	0.091	0.001	0.088	0.007	0.006	0.088	0.088	–	2	60	70	2	61	59	107	97	97	118	114	71	72	72
10	*Glugea nagelia*	0.303	0.093	0.001	0.089	0.007	0.006	0.089	0.089	0.003	–	61	71	2	62	60	108	98	98	119	115	73	72	74
11	*Glugea plecoglossi*	0.311	0.001	0.088	0.001	0.085	0.091	0.001	0.001	0.089	0.091	–	17	60	3	1	102	88	90	107	116	73	71	77
12	*Glugea sardinellensis*	0.325	0.027	0.105	0.024	0.101	0.107	0.024	0.024	0.106	0.107	0.026	–	70	18	16	113	100	101	118	126	84	82	88
13	*Glugea serranus*	0.301	0.091	0.001	0.088	0.007	0.006	0.088	0.088	0.003	0.003	0.089	0.106	–	61	59	108	98	98	118	114	72	71	73
14	*Glugea stephani*	0.313	0.006	0.089	0.003	0.086	0.092	0.003	0.003	0.091	0.092	0.004	0.027	0.091	–	2	103	89	91	109	118	76	72	79
15	*Glugea thunni* sp. nov	0.313	0.003	0.086	0.000	0.083	0.089	0.000	0.000	0.088	0.089	0.001	0.024	0.088	0.003	–	101	87	89	107	116	74	70	77
16	*Loma embiotocia*	0.322	0.154	0.158	0.150	0.159	0.164	0.150	0.150	0.159	0.161	0.152	0.171	0.161	0.153	0.150	–	20	13	142	140	123	123	128
17	*Loma morhua*	0.325	0.133	0.145	0.129	0.145	0.149	0.129	0.129	0.145	0.146	0.131	0.151	0.146	0.132	0.129	0.030	–	7	131	131	115	112	119
18	*Loma salmonae*	0.324	0.136	0.145	0.132	0.145	0.149	0.133	0.132	0.154	0.146	0.134	0.153	0.146	0.135	0.132	0.019	0.010	–	134	132	113	112	117
19	Microgemma caulleryi*	0.307	0.169	0.184	0.167	0.185	0.188	0.166	0.167	0.185	0.187	0.167	0.188	0.185	0.170	0.167	0.222	0.205	0.210	–	50	120	115	124
20	*Microgemma* sp. partial 16S	0.298	0.182	0.175	0.179	0.179	0.181	0.177	0.179	0.176	0.177	0.179	0.197	0.176	0.182	0.179	0.216	0.202	0.204	0.079	–	128	118	128
21	*Pleistophora ehrenbaumi*	0.319	0.113	0.109	0.112	0.106	0.112	0.112	0.112	0.107	0.110	0.11	0.129	0.109	0.115	0.112	0.186	0.173	0.171	0.190	0.200	–	53	56
22	*Pleistophora mirandellae*	0.331	0.106	0.105	0.104	0.108	0.111	0.104	0.104	0.108	0.108	0.106	0.124	0.106	0.107	0.104	0.184	0.167	0.167	0.180	0.182	0.080	–	5
23	*Pleistophora typicalis*	0.323	0.117	0.110	0.115	0.106	0.112	0.115	0.115	0.108	0.111	0.115	0.134	0.109	0.118	0.115	0.192	0.178	0.175	0.195	0.198	0.084	0.008	–

*Accepted as *Glugea microspora* inLom (2002)

**Table 3:** Differences among representatives of the genera *Brachiola*, *Glugea*, *Loma* and *Pleistophora* for 16S rDNA sequences, pairwise nucleotide differences (above the diagonal) and p-distances (below the diagonal) 1713 bp sequences

	Species	–1	2	3	4	5	6	7	8	9	10
1	*Brachiola algerae*	–	479	471	470	473	474	473	474	501	493
2	*Glugea anomala*	0.320	–	152	149	17	155	151	14	242	205
3	*Glugea arabica*	0.314	0.097	–	16	142	12	11	142	231	182
4	*Glugea eda*	0.315	0.096	0.010	–	139	12	7	139	230	176
5	*Glugea hertwigi*	0.316	0.011	0.091	0.089	–	145	141	7	234	200
6	*Glugea nagelia*	0.316	0.099	0.008	0.008	0.092	–	7	145	235	175
7	*Glugea serranus*	0.315	0.096	0.007	0.004	0.090	0.004	–	141	231	174
8	*Glugea thunni* sp. nov	0.316	0.009	0.091	0.089	0.004	0.092	0.090	–	236	197
9	*Loma embiotocia*	0.334	0.155	0.148	0.148	0.149	0.150	0.147	0.151	–	277
10	*Pleistophora ehrenbaumi*	0.330	0.131	0.117	0.113	0.128	0.112	0.111	0.126	0.177	–

### Taxonomy

*Glugea thunni* López-Verdejo A., Montero F.E., de la Gándara F., Gallego M.A., Ortega A., Raga J. and Palacios-Abella J.F., sp. nov.

*Etymology*: The species epithet refers to *Thunnus*, the genus of the type host species.

*Diagnosis*: This species can be distinguished from other congeneric species by the combination of morphological traits such as spore measurements and number of polar filament coils, and the new host species (and family) for the genus *Glugea*. The new species can be distinguished from the other microsporidians in *Thunnus* spp. by: (1) the infection site (mesenteries of caecal mass and viscera for *G. thunni vs*. trunk muscle and intestinal muscularis mucosa for *Microsporidium* sp. and *M. milevae* respectively; (2) xenoma traits (subspherical/to 7.5 mm *vs* spindle-shaped/to 6 mm and spherical-elongated/2.1 × 0.8 mm); (3) spore traits in fresh (ovoid to ellipsoidal/2.0–2.5 × 3.6–4.5 μm *vs*. oval to pyriform/2.4–2.9 × 1.2–1.7 μm and pyriform/2.45 ± 0.28 × 4.88 ± 0.31 μm); and (4) polar filament arrangement in spores (13–14 coils in single row in *G. thunni vs.* 12–17 coils in two rows in *M. milevae*; not indicated in *Microsporidium* sp.) (Zhang et al. 2004; Mladineo and Lovy 2011).

*Type*: Spain: *Murcia*: sea cage off San Pedro del Pinatar, 37°49′ 32.0″ N 0°44′54.3″ W, on a 5 month old hatchery-reared juvenile of *Thunnus thynnus* (*Perciformes*, *Scombridae*), Nov. 2017, (MNCN:ADN:119975—holotype [Histological resin and paraffin sections]); MNCN:ADN:119976—paratype [ICZN] = isotype [ICNapf]). Additional paratype material is deposited in the Parasitological Collection, Cavanilles Institute of Biodiversity and Evolutionary Biology, University of Valencia, Spain. Representative 16S rDNA (1751 bp) sequences uploaded to GenBank under Accession no. OM914139.

**Fig. 1:**
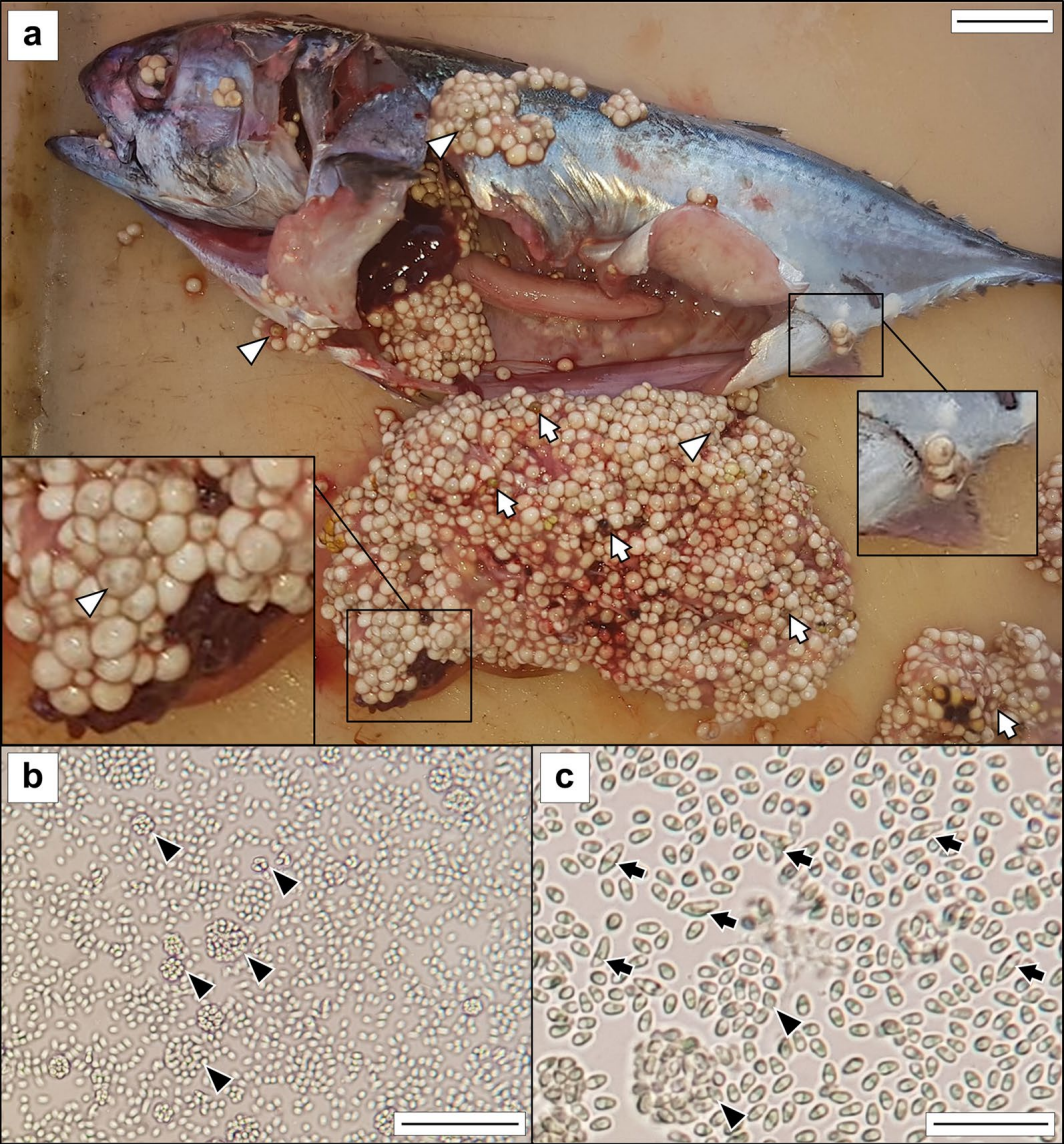
*Glugea thunni* in *Thunnus thynnus* from the Mediterranean Sea. **a** Specimen of *T. thynnus* infected by *G. thunni* with melanized and partially melanized cysts, including a detail of xenomas within the ceacal mass (scale bar 2.5 cm) **b** fresh smear with free microspores and parasitophorous vesicles with different number of microspores (scale bar 40 µm). **c** Detail of fresh smear with free short and large microspores and parasitophorous vesicles (scale bar 20 µm). White arrow—melanized cyst; white arrowhead—cysts with melanized spots; black arrows—abnormal microspores; black arrowheads—parasitophorous vesicles

**Fig. 2:**
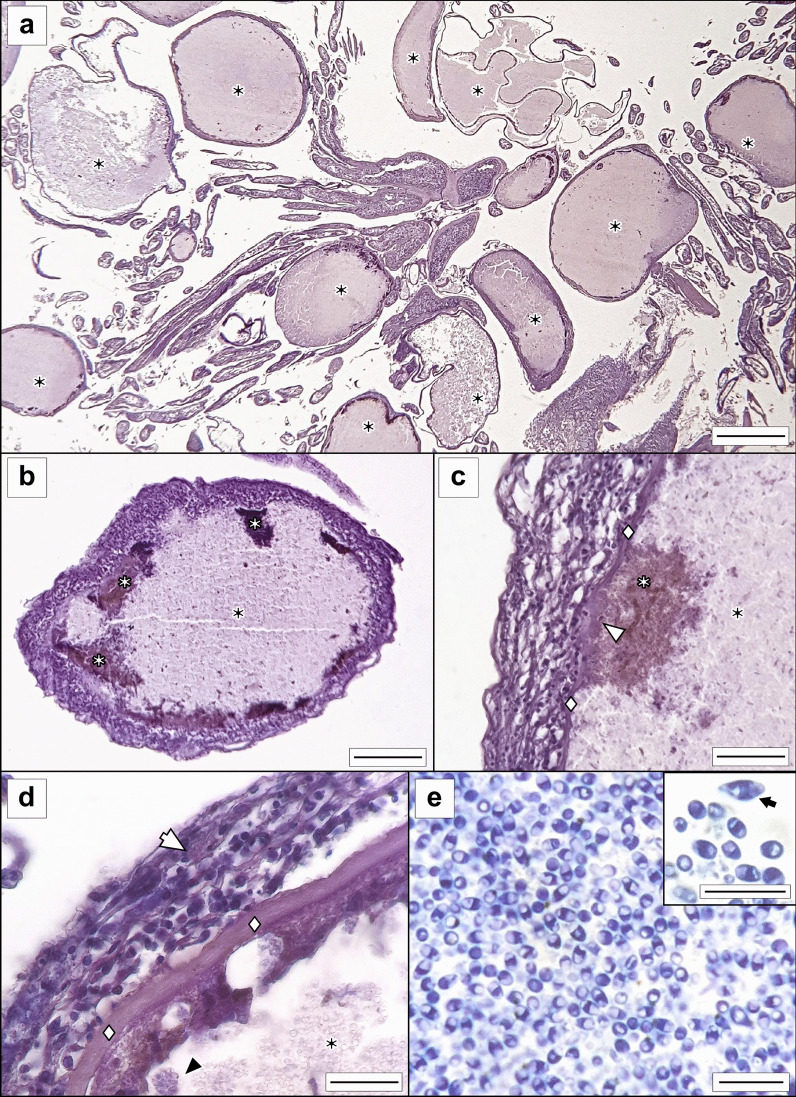
Micrographs of *Glugea thunni* from histological sections of the caecal mass of *Thunnus thynnus* from the Mediterranean Sea. **a** *G. thunni* xenomas in the mesentery among the intestinal caeca (scale bar 1 mm). **b** Cyst of *G. thunni*; xenoma exhibits peripheral spots with different degrees of melanization (scale bar 200 µm). **c** Detail of peripheral xenoma melanization (scale bar 70 µm). **d** Cyst wall with eosinophilic granule cell in the outer celular layer (scale bar 40 µm). **e** Microspores at the central region of xenoma (scale bar 10 µm) with a detail including an abnormal microspore (scale bar 5 µm) (**a**–**d**, paraffin sections stained in H–W; e, semi-thin stained in toluidine blue). White arrow—eosinophilic granule cell; black asterisk—xenoma; white asterisk—melanized spot; white diamond—acellular/fibrous layer; white arrowhead—disintegrated acellular/fibrous layer; black arrowhead—parasitophorus vesicle

**Fig. 3:**
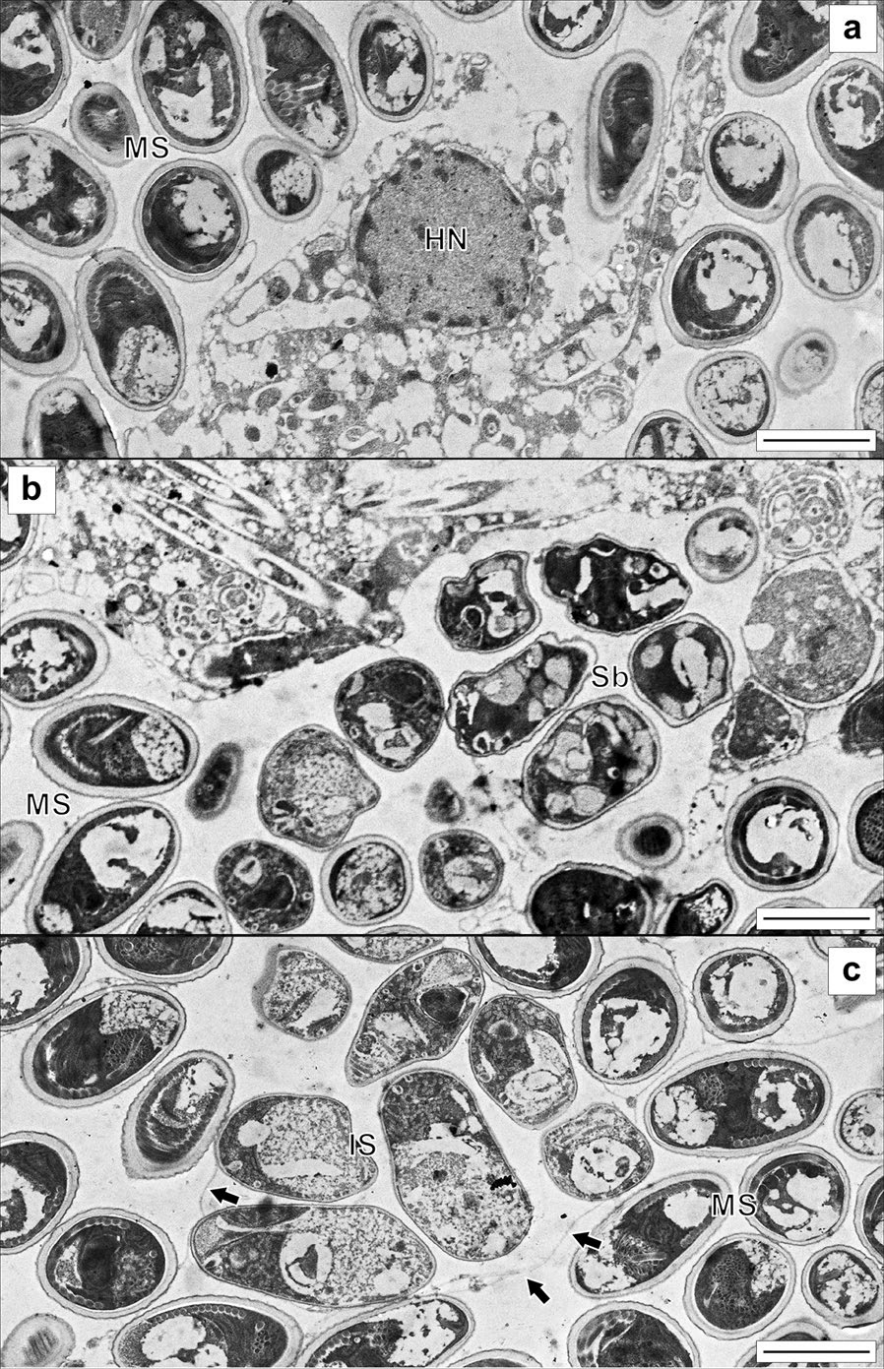
Transmission electron micrographs of xenomas of *Glugea thunni* from the caecal mass of T*hunnus thynnus* from the Mediterranean Sea. **a** Mature spores and degenerative host cells. **b** and **c** Spores under development. MS—mature spores; IS—immature spores; Sb—Early sporoblast; HN, Host cell nucleus; black arrowhead—parasitophorous vacuole membrane (scale bars 2 µm)

**Fig. 4:**
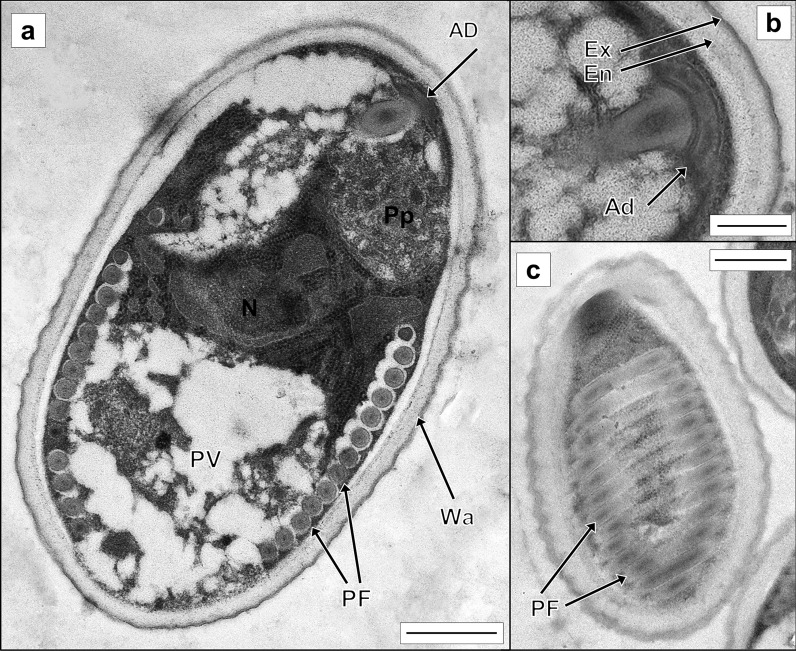
Transmission electron micrographs of *Glugea thunni* from the caecal mass of *Thunnus thynnus* from the Mediterranean Sea. **a** Longitudinal section of an adult microspore showing the ultrastructure (scale bar 500 nm). **b** Detail of the anchoring disk and spore wall (scale bar 400 nm). **c** Detail of polar filament surrounding the spore (scale bar 1 µm). Abbreviations: AD—anchoring disk; En—wall endospore; Ex—wall exospore; N—spore nucleus; PF—polar filament; Pp—polaroplast; PV—posterior vacuole; Wa—spore wall

*Description*: *Xenomas* whitish, subspherical to ellipsoidal, in cysts mostly associated to the caecal mass, 0.2 to 7.5 mm diam, with an average size of 3.3 mm (*n* = 40); some xenomas also in the liver, peritoneum and cloaca (Fig. 1A). In fresh smears, spores arranged within parasitophorous vesicles (in groups of approx. 3–100, Fig. 1B–C). *Spores* ovoid to ellipsoidal in shape, 2.2 × 3.9 (2.0–2.5 × 3.6–4.5) μm (*n* = 10) in fresh (light microscopy); some larger spores, 2% approximately (2.4 × 6.5 (1.6–2.7 × 6.0–6.8) μm (*n* = 10) were also found, elongated and/or bent and fusiform in shape (Figs. 1C, 2E). In semi-thin sections, ovoidal spores measuring 2.1 × 3.8 (1.9–2.5 × 3.5–4.0) μm (*n* = 10) and large fusiform spores 2.6 × 6.2 (2.5– 2.7 × 5.9–6.4) μm (*n* = 10) (Fig. 2E). In TEM, xenomas with numerous spores enclosed within parasitophorous vacuoles with faint membranes together with degenerative host cells (Fig. 3A). Mature spores in TEM images measuring 2.22 × 3.62 (1.82–2.44 × 3.14–4.26) μm (*n* = 10). *Developmental stages* (Fig. 3B, C): early sporoblasts irregular and thin-walled whilst immature spores ovoid to fusiform, somewhat larger than mature spores (2.38 × 5.16 (2.01–2.28 × 4.11–5.36) μm; *n* = 3) with a well-defined spore wall, although smooth and thinner. Mature spores ovoid with a rugous surface. Wall of mature spores double-layered, formed by an electron-lucent endospore and a thin electron-dense exospore with an altogether thickness of 0.113 (0.079–0.158) μm (Fig. 4A, B). Nucleus, irregular in shape, medial, between posterior vacuole and apical polaroplast (Fig. 4A). Posterior vacuole occupying most of the second half of the mature spore, almost completely surrounded by the polar filament with 13–14 coils in one single layer (Fig. 4A, C) ending in a subapical concave anchoring disc (0.320–0.351 μm diam, 0.077–0.078 thickness; *n* = 2) (Fig. 4B).

*Host*: *Thunnus thynnus*.

*Habitat*: Aquaculture sea-cage off San Pedro del Pinatar, Murcia, Spain.

*Distribution:* Western Mediterranean.

## RESULTS

### Clinical signs and diagnosis

The infected fish was found freshly dead with a conspicuously swollen abdomen (Fig. 1A). The rest of the tuna in the routine control did not show this alteration. The intestinal zone of the abdominal cavity was occupied by highly numerous whitish xenomas. some of them showed up externally (see detail in Fig. 1A). Several xenomas showed melanization in dark brownish or yellowish spots. In sections, xenomas were found in the intestinal mesentery, in clusters associated to the caecal mass (Fig. 2A). Xenomas appeared encapsulated by a layer of host cells, with spores and sporoblasts grouped within parasitophorous vacuoles together with degenerative host cells (nuclei observed in TEM, see Fig. 3A). Cyst walls had an external host cell layer and an internal acellular layer; both layers showed different thickness in each xenoma (see Fig. 2B, D). Eosinophylic granule cells and macrophages were observed within the cellular layer (Fig. 2D). Several xenomas exhibited peripheral areas of melanization (Fig. 2B–C).

### Molecular and phylogenetic analysis

A 16S rDNA sequence of 1751 bp was obtained for the new *Glugea* specimens and then compared to the database sequences from Genbank with the BLAST tool. The most similar sequences to the new one here obtained were AF044391 (*G. anomala*) and GQ203287 (*G. hertwigi*) with 100% query coverage and a similarity of 97.91% (23 bp of difference) and 99.09% (13 bp of difference) respectively, showing very low differences among species. Tables 2 and 3 show the p-distances and differences of pair bases among the sequences used in the performed alignments. In the first rDNA alignments 774 informative positions were included (short sequences) comprised of 22 sequences in the ingroup with *Brachiola algerae* (Coyle et al. 2004) used as outgroup. Due to the short length of the trimmed sequences, the differences of bp between some of the sequences was 0 (Table 2).

**Fig. 5:**
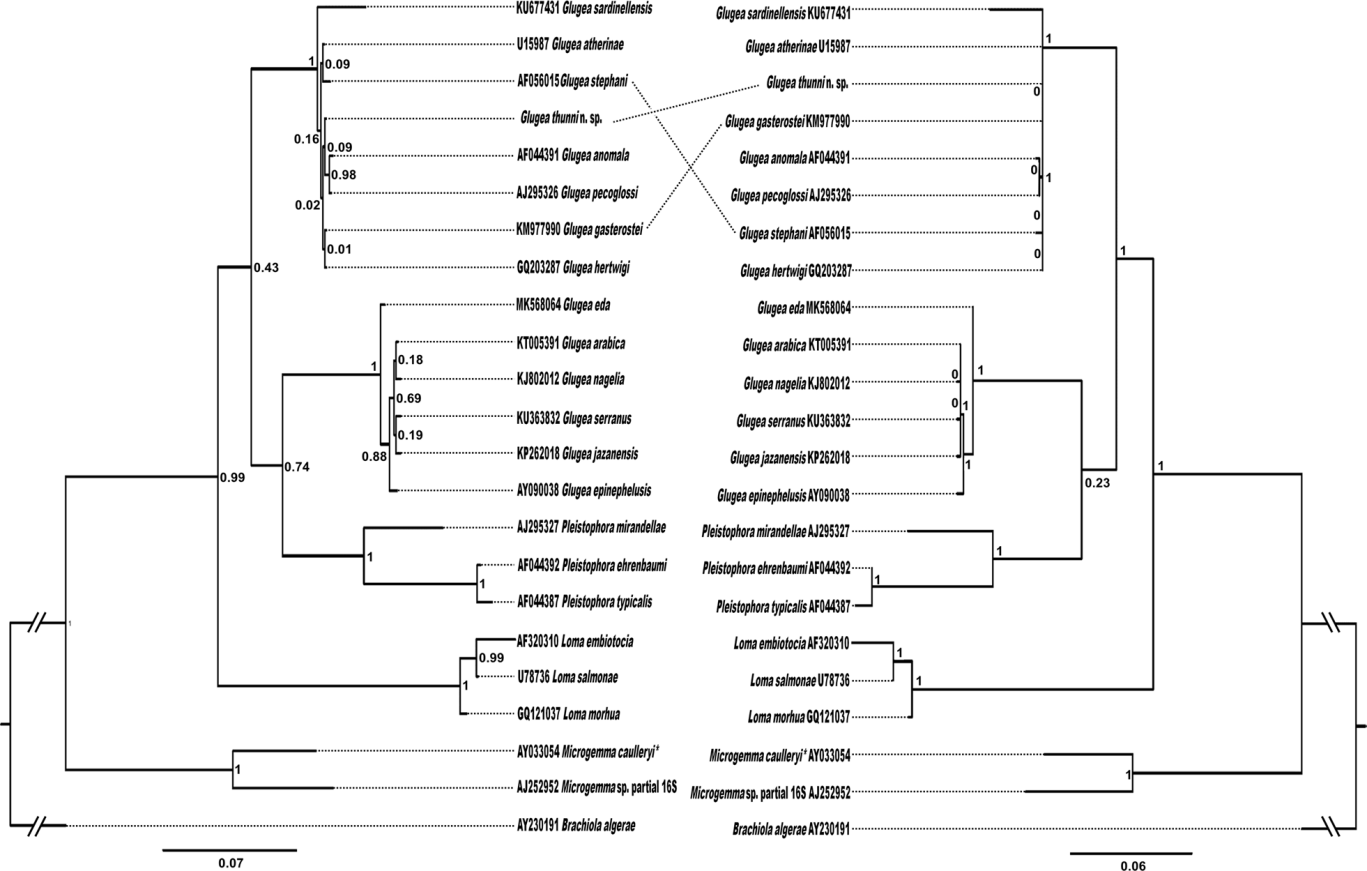
Confronted bayesian inference (BI) and maximum likelihood (ML) trees for the analyses of the microsporidians based on partial 16S rDNA sequences (774 bp). Nodal support is given as posterior probabilities (BI) and bootstrap values resulting from maximum likelihood (ML); only values > 0.95 (BI) and 70% (ML) are shown. The scale-bars indicate the expected number of substitutions per site. *Microgemma caulleryi is accepted as *G. microspora* in Lom 2002

The result of BI and ML from this alignment solved the trees in the same way but the lack of support in the lower relationships must be highlighted. In both BI and ML there is a basal clade formed by *Microgemma* species (partial 16S), then the next clade that separates is the one formed by *Loma* species. Afterwards we can observe a bifurcation from which two groups appear. In the first group there is one clade formed by *Pleistophora* species and a second clade made up of six *Glugea* species (*G. arabica*, *G. eda*, *G. epinephelusis*, *G. jazanensis*, *G. nagelia*, and *G. serranus*), species in Group 2 sensu (Mansour et al. 2016) (G2). The second group is made up of the remaining *Glugea* species (*G. anomala*, *G. atherinae*, *G. gasterostei*, *G. hertwigi*, *G. plecoglossi*, *G. sardinellensis* and *G. stephani*), species in Group 1 sensu (Mansour et al. 2016) (G1) including the new *Glugea thunni* (Fig. 5).

**Fig. 6:**
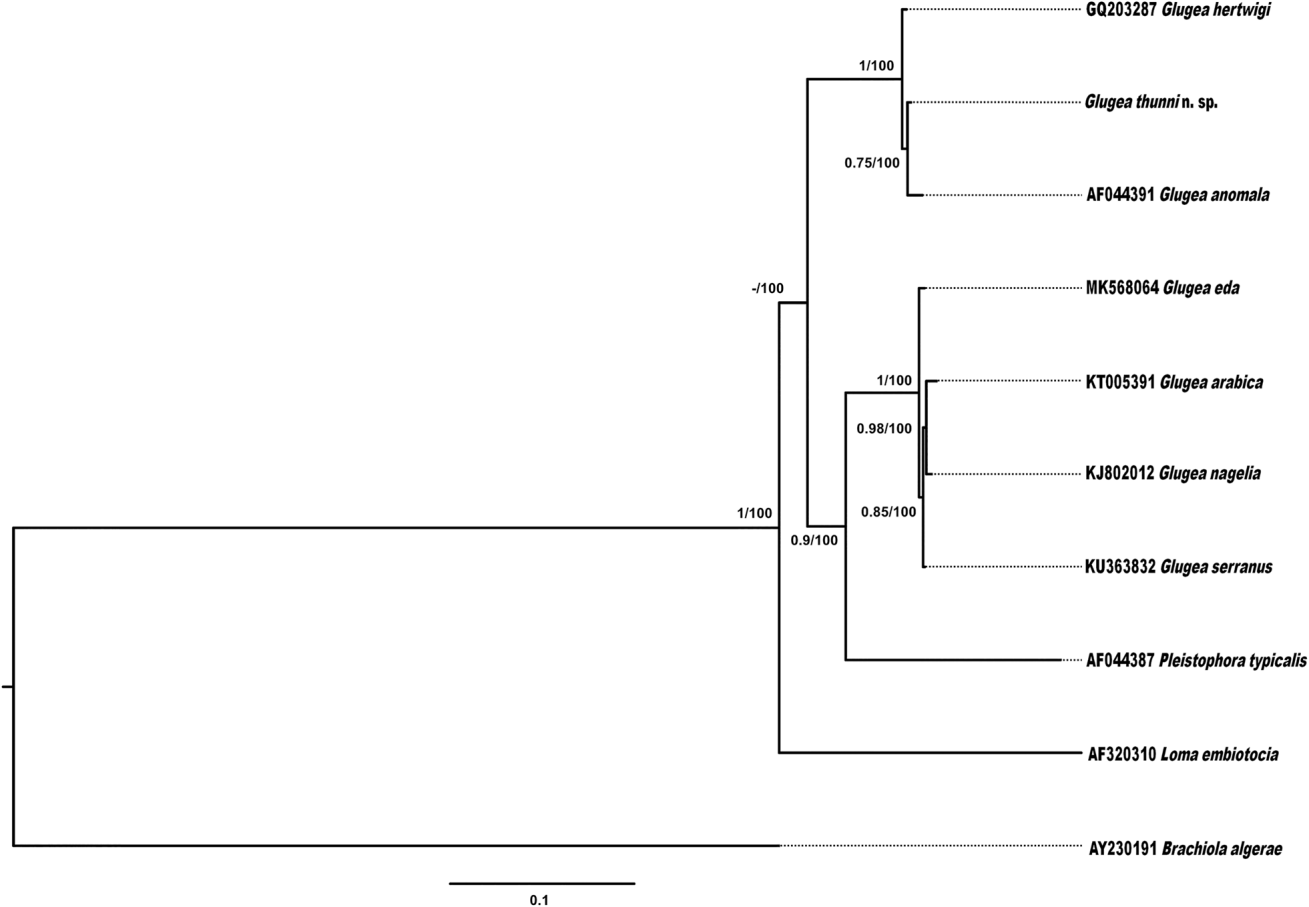
Resulting tree for the analyses of the microsporidians based on partial 16S rDNA sequences (1713 bp). Nodal support is given as posterior probabilities (BI) and bootstrap values resulting from maximum likelihood (ML) analysis in the form (BI/ML); only values > 0.95 (BI) and 70% (ML) are shown. The scale-bar indicates the expected number of substitutions per site

The second alignment was performed with longer sequences (1713 informative positions), including nine sequences in the ingroup and *B. algerae* (Edgar 2004) as outgroup. By using these longer sequences, differences in bp not observed in the previous shorter alignment were now shown, such as between *G. hertwigi* and *G. thunni* (Table 3). In the same way, both BI and ML resulted in similar tree, although in this case high nodal supports were obtained, in contrast with the previous trees that had more taxa but shorter sequences. The first taxa that diverges is *L. embiotocia*, then two groups, one formed by and *Pleistophora typicalis* basal to *G. arabica*, *G. eda*, *G. nagelia* and *G. serranus* and the second one formed by *G. hertwigi*, *G. anomala*, and *G. thunni* (Fig. 6).

## DISCUSSION

Currently, 35 species of *Glugea* have been described (Azevedo et al. 2016; Mansour et al. 2020). *Glugea thunni* possess the morphological traits of the genus *Glugea *sensu Lom (2002): unpaired nuclei throughout development, thin membrane-like wall of parasitophorous vesicle, monomorphic mature spores and isofilar polar tube coiled in single row. This diagnosis would include the new described species in the subclade G1 described by Mansour et al. (2016) including mostly Mediterranean parasites. However, Lom’s generic description would not include the six congeneric species more recently described, mostly from the Red Sea and Arabian Gulf, included in the subclade G2 (Mansour et al. 2016), in which polar tubes are arranged in several rows (this trait not described in *G. epinephelusis*) (Zhang et al. 2004; Wu et al. 2005; Mansour et al. 2016). Within the subclade G1, other similar species to the new *Glugea* species are *G. anomala*, *G. gasterostei*, *G. hertwigi*, *G. plecoglossi*, *G. sardinellensis* and *G. stephani*, based on the range of number of coils and the spore width range; however, the spore of *G. thunni* is shorter in mean measurements (Canning et al 1982; Takvorian and Cali 1983; Takahashi and Egusa 1977a; Lovy et al. 2009; Tokarev et al. 2015, Mansour et al. 2016). Within this group, the most similar species is *G. sardinellensis* with a similar spore shape and the same range of number of coils (13–14): however, the new species is different from *G. sardinellensis* by the above-mentioned shorter spores and the much larger maximum size of the xenomas (probably related with the host size: *T. thunnus vs. Sardinella aurita*).

Regarding the molecular results from both phylogenetic trees with long and short sequences, the distribution of the *Glugea* species in the present study were identical to the ones observed in Mansour et al. (2016). *Glugea thunni* is included in the G1 group cited above, which is congruent with the morphological similarity. However, the relationships between species within this G1 group are not well resolved due to the short sequences available and the low genetic divergences obtained in the SSU-LSU genes (Fig. 5; Table 2). The two closest species genetically to *G. thunni* are *G. hertwigi* from the intestine of *Osmerus epperlanus,* and *G. anomala* from the muscle of *Gasterosteus aculeatus*. Low but significant differences among these species are observed only by using longer sequences of *G. anomala*, *G. hertwigi,* and *G. thunni* (used in the second alignment of present work): p-distances range from 0.4% of differences between *G*. *thunni vs. G. hertwigi*; 0.9% between *G. thunni vs G. anomala*; and to 1.1% from *G. hertwigi vs*. *G. anomala* (Fig. 6; Table 3). The phylogenetic tree resulting from the long sequences revealed *G. anomala* as the closest species to *G*. *thunni*, instead of *G. hertwigi*. Surprisingly, contrary to the morphological information, *G. sardinellensis* was the most distant species to *G. thunni* among those of the G1 group with at least 2.4–2.7% of differences in respect to their other relatives (Fig. 5; Table 2).

Despite the genetical differences and the different host species, the morphology of *Glugea thunni* is very similar to *G. sardinellensis*, *G. anomala* and *G. hertwigi*. *Glugea sardinellensis* is the morphologically closest regarding number of coils of the polar filament (13–14 for both species), however the spore is longer (2.75 × 5.25 (2.5–3.0 × 5.0–5.5) vs. 2.1 × 3.8 (1.9–2.5 × 3.5–4.0) in *G. thunni*). The morphology of the spores of *G. anomala* and *G. hertwigi* is almost indistinguishable: *G. anomala* and *G. hertwigi* have slightly longer spores (2.3 × 4.6 (1.9–2.7 × 3.0–5.6) and 2.4 × 5.4 (2.1–2.6 × 4.8–6.0) respectively vs. 2.1 × 3.8 (1.9–2.5 × 3.5–4.0) in *G. thunni*); also the spores have 13–15 and 12–13 polar filament coils respectively, arranged in one single layer, compared with 13–14 of *G. thunni* arranged in one single layer too. Host species seems the most reliable character to identify these species.

An additional sequence labelled as “*G. plecoglossi*” (KY882286, unpubl.) exists in GenBank. This microsporidian could have been inaccurately identificated, as its sequence is different to those of *G. plecoglossi* from other studies but almost identical to *G. thunni*. In the absence of morphological confirmation, molecular results indicate that “*G. plecoglossi*” (KY882286) and *G. thunni* could be the same species. This information could be useful to determine the transmission path in ABT cultures, as “*G. plecoglossi*” (KY882286) was found in a clupeid (*Sardina pilchardus*, *Clupeidae*), a fish that is commonly used as bait to feed tuna in the Spanish farms (e.g. *Sardinella aurita*, *Clupeidae.* The other microsporidian species known from bluefin tunas, *Microsporidium* sp. and *M. milevae* (Zhang et al. 2004, Mladineo and Lovy 2011), are not included in these comparisons as they are not genetically or morphologically close.

In recent years, molecular data has become an essential tool for taxonomical analyses of the microsporidia, however most of the species are only characterized by their ultrastructure, xenoma traits, host specificity or life cycle (Corradi and Keeling 2009; Azevedo et al 2016). The fact that only 14 sequences of *Glugea* spp. (including *G. thunni*) are available in GenBank makes it necessary to combine molecular analyses and other biological traits to elucidate the phylogenetic relationships of the microsporidians. In this context, the morphological and molecular classifications must be congruent. Several *Glugea* species recently described, which have been genetically included in G2 according to Mansour (2016), do not exhibit one of the diagnostic traits of the genus, the arrangement of polar tubes in a single row (Lom 2002); there are several rows in G2. Based on the different morphology and the separation of G1 and G2 in the phylogenetic trees, the inclusion of G2 within *Glugea* seems doubtful. We also strongly recommend obtaining longer sequences, with similar coverage, in order to obtain more solid results to clarify the phylogenetic relationships among this diverse parasite group.

According to Azevedo et al. (2016), the species of *Glugea* have a preference either for smooth musculature or connective tissues of visceral organs. *Glugea thunni* shares this habitat preference with *G. hertwigi*, one of the phylogenetically closest species (Lovy et al. 2009). The infection of visceral mesenteries in this investigation allowed a wide parasite dispersion, not only in the caecal mass, but also in other intestinal regions and the liver; moreover, this extensive infection had to have been achieved in a relatively short time, due to the young age of the specimen (five months). The impact of this parasite seems different to that of the other microsporidians in bluefin tunas; *Microspora* sp. was reported in the muscle of *T. orientalis* (Zhang et al 2010), which could affect product value, while *M. milevae* infects the muscularis mucosa of *T. thynnus* (Mladineo and Lovy 2011), which could affect the intestinal function. However, these *Microspora* spp. infections seem more localized than that which is associated to *G. thunni* sp. nov., and therefore their consequences appear to be milder. Moreover, the massive alterations of viscera associated with *G. thunni* sp. nov. is likely to cause rejection by the consumer.

The new species shows a high capability to spread within the host, reaching a high intensity, however, the parasite was found in only one fish of the sea cage. Transmission of fish microsporidians is described as trophic and direct, although some crustaceans could also take part in the life cycle (Lom and Nilsen 2003; Lom and Dyková 2005). In aquaculture conditions, a small number of zooplankton can reach sea cages, but the most probable infection path of the parasite is through bait or by cannibalism. The transmission capability of these parasites among different tunas has been quite limited, however, in view of the severe consequences of the parasite, prevention measures are needful. The removal of dead fish is highly recommended, as well as, when possible, ill and moribund fish. Nonetheless, infected food appears to be the main issue to deal with this disease, as it is the most probable pathway for this parasite to have entered in the cultures, as tunas of this study were not captured from the wild for fattening. These ABTs were born in captivity and fed with thawed bait, mostly clupeids. *Glugea thunni* could also infect clupeids as although the type host is *T. thynnus*, clupeids are frequent hosts of *Glugea* spp. (Mansour et al. 2016) and, more importantly, the new species sequence is the same as KY882286 in GenBank, an unpublished sequence apparently wrongly identified as “*G. plecoglossi*” from *Sardina pilchardus* (*Clupeidae*). Therefore, an adequate management of the bait is highly recommendable. Bait is routinely frozen (approx. − 18 °C) to avoid horizontal transmission of anisakid nematodes, an important concern for consumer health. This process would also affect *G. thunni* infectivity. The development of *G. plecoglossi* is known to be slowed at − 16 °C (Takahashi and Egusa 1977b) and *G. stephani* experimental infection failed at − 15 °C (Olson 1976). However, it is known that some microsporidians show a high resistance to low temperatures (up to − 80 °C) (Maddox and Solter 1996). The harshness of this parasite makes it necessary to study its viability at low temperature.

## CONCLUSION

This is the first report of a microsporidian disease in cultured Atlantic bluefin tuna, despite other species have been reported in other *Thunnus* species. It is important to properly identify the microsporidian species for a correct management of the disease, since each species has a different biology, with different hosts involved in their life cycles, meaning that the entry routes of the parasite can be very different. Nowadays, accurate morphological and molecular differentiation among *Glugea* species is challenging, therefore, it is necessary to increase the available knowledge of this group, principally by boosting the quantity and quality of the accessible genetic sequences.

It is important to highlight the potential degree of damage of this microsporidian could cause in marine aquaculture systems of Atlantic bluefin tuna, one of the most expensive and appreciated fish worldwide. Nowadays, there are no effective treatments against microsporidia in fish, except for some sporadic and inconclusive reports (toltrazuril for *G. anomala* and fumagilin for *G. plecoglossi*; Fleurance et al. 2008). Other fungicides or new therapeutic strategies to control microsporidian diseases are needed. Thus, prevention appears to be the most recommendable way to cope with the disease, which requires a knowledge of the transmission paths. Future investigations should therefore focus on: (1) searching for the parasites in clupeids of bait to determine their role as possible disease entry; (2) studying the effect of low temperatures in the microsporidian infectivity; and (3) finding alternative ways to treat the food to inactivate the parasite. Despite the lack of crucial information, avoiding dangerous practices as the use of fresh and never frozen bait is highly recommendable to prevent this disease, especially when clupeid fishes are used as food.

## Data Availability

All data generated or analyzed during this study are included in this published article. The newly generated sequence was submitted to the GenBank database under the accession number OM914139. The holotype and paratype of *G. thunni* have been deposited in the Spanish Museum of Natural Sciences (MNCN-CSIC), Madrid, Spain (MNCN:ADN:119975 and 119976); the remaining material is deposited in the Parasitological Collection of the Cavanilles Institute of Biodiversity and Evolutionary Biology, University of Valencia, Spain.

## Abbreviations

ABT: Atlantic bluefin tuna (*Thunnus thynnus*)
AD: Anchoring disk
En: Wall endospore
Ex: Wall exospore
HN: Host cell nucleus
IS: Immature spores
MM: Mature microspores
MS: Mature spores
N: Spore nucleus
PF: Polar filament
Pp: Polaroplast
PV: Posterior vacuole
Sb: Early sporoblast
Wa: Spore wall
